# A Novel Strategy to Study the Invasive Capability of Adherent-Invasive *Escherichia coli* by Using Human Primary Organoid-Derived Epithelial Monolayers

**DOI:** 10.3389/fimmu.2021.646906

**Published:** 2021-03-29

**Authors:** Aida Mayorgas, Isabella Dotti, Marta Martínez-Picola, Miriam Esteller, Queralt Bonet-Rossinyol, Elena Ricart, Azucena Salas, Margarita Martínez-Medina

**Affiliations:** ^1^ Department of Gastroenterology, IDIBAPS, Hospital Clínic, CIBER-EHD, Barcelona, Spain; ^2^ Laboratory of Molecular Microbiology, Department of Biology, Universitat de Girona, Girona, Spain

**Keywords:** organoid-derived epithelial monolayers (ODM), adherent-invasive *E. coli* (AIEC), bacterial infection, intestinal epithelial cells (IECs), inflammatory bowel disease (IBD)

## Abstract

Over the last decades, Adherent-Invasive *Escherichia coli* (AIEC) has been linked to the pathogenesis of Crohn’s Disease. AIEC’s characteristics, as well as its interaction with the gut immune system and its role in intestinal epithelial barrier dysfunction, have been extensively studied. Nevertheless, the currently available techniques to investigate the cross-talk between this pathogen and intestinal epithelial cells (IECs) are based on the infection of immortalized cell lines. Despite their many advantages, cell lines cannot reproduce the conditions in tissues, nor do they reflect interindividual variability or gut location-specific traits. In that sense, the use of human primary cultures, either healthy or diseased, offers a system that can overcome all of these limitations. Here, we developed a new infection model by using freshly isolated human IECs. For the first time, we generated and infected monolayer cultures derived from human colonic organoids to study the mechanisms and effects of AIEC adherence and invasion on primary human epithelial cells. To establish the optimal conditions for AIEC invasion studies in human primary organoid-derived epithelial monolayers, we designed an infection-kinetics study to assess the infection dynamics at different time points, as well as with two multiplicities of infection (MOI). Overall, this method provides a model for the study of host response to AIEC infections, as well as for the understanding of the molecular mechanisms involved in adhesion, invasion and intracellular replication. Therefore, it represents a promising tool for elucidating the cross-talk between AIEC and the intestinal epithelium in healthy and diseased tissues.

## Introduction

*Escherichia coli* (*E. coli*) strains are widely known inhabitants of the healthy human gut microbiota, being one of the first colonizers as well as among the most prevalent microorganisms in the intestines (1, 2). *E. coli* promotes health benefits to its hosts by preventing the colonization of pathogens and thus, positively contributes to intestinal homeostasis (3, 4). However, several *E. coli* strains, including the Adherent-Invasive *E. coli* (AIEC) pathotype, have acquired a virulent nature. Despite the lack of typical enteropathogenic *E. coli* virulent factors in AIEC isolates, these are able not only to adhere to and invade intestinal epithelial cells (IECs), but also to replicate within macrophages without inducing cell death, thus evading protective host immune responses (5–7).

AIEC was first identified in the ileal mucosa of patients with Crohn’s Disease (CD) and may constitute more than the 50% of the total number of bacteria both in early and chronic ileal lesions (8, 9). AIEC prevalence in Inflammatory Bowel Disease (IBD) – which comprises CD and Ulcerative Colitis (UC) – patients is significantly higher than in non-IBD subjects and, in general, AIEC strains are found in ileal and colonic samples of CD patients (6, 10–17). In UC, although the prevalence of this pathobiont is less clear, a recent meta-analysis suggests that this pathotype could be involved in its pathogenesis (18). Both *in vitro* and *in vivo* assays helped explain the molecular basis of AIEC pathogenicity in CD (9, 19). AIEC mechanisms to cross the mucus layer include the secretion of bacterial proteases (20, 21) as well as the alteration of host antimicrobial peptides (22). Adhesion and invasion to IECs occurs through the interaction between, among others, AIEC type 1 pili and the eukaryotic glycoprotein CEACAM6 (23, 24). On the other hand, flagella are crucial in mediating AIEC-induced cellular responses through their binding to IECs-toll like receptor (TLR)-5 (25). All these events end up triggering a cytokine release which, in turn, promotes intestinal epithelial permeability (26) and intestinal inflammation in compromised patients (27). AIEC are also able to invade M cells and translocate through Peyer’s patches reaching the lamina propria and rapidly spreading through the mesenteric lymph nodes (28–30), and to translocate across the intestinal barrier due to tight junctions expression alteration (31). Overall, it has been demonstrated that AIEC infections affect a wide variety of host cell processes such as protein synthesis, signal transduction, cell division, and cytoskeletal function among many others (32).

AIEC identification is currently challenging, as it relies on phenotypic assays based on infected cell cultures, which are highly time-consuming. Therefore, the identification of AIEC molecular markers is of great importance since it would support detection of AIEC carriers, which is necessary to carry out epidemiological studies and to eventually establish prevention protocols (33–35). Different immortalized cell lines have been applied to assess the AIEC phenotype. The most common ones for the study of AIEC adhesion and invasion capacity are Caco2, Intestine-407 (I407), T84 and Hep2 as reviewed by Camprubí-Font et al. (36). Even though cell lines are easy to obtain, handle and expand over time, they lack important physiological features such as tissue cytoarchitecture, inter-individual variability and gut location-specific attributes. All of these limitations can be overcome by using human primary cultures. Organs or tissues isolated from their *in vivo* environment offer the advantage of providing a more physiological experimental setting due to their mimesis of the tissue of origin, phenotype and structure. Hence, infecting human colonocytes derived from patient biopsies might represent a promising strategy for studying the intestinal epithelium response to AIEC, as well as new pathogenicity mechanisms associated with this pathobiont. Such an approach could lead to the discovery of new disease biomarkers and new therapeutic targets. To our knowledge, there are few publicly available reports that analyze the interaction between enteric pathogens and human isolated IECs (23, 37–41). More recently, Sayed et al. published a study in which AIEC infection of organoid-derived 2D cultures is applied to explore host engulfment in IBD. Their research supports the suitability of human organoid-derived epithelial monolayers (ODMs) as a tool to study AIEC pathogenicity (42). Here, we deeply describe our recently developed infection method that uses colonic ODMs to examine the ability of AIECs to adhere to and invade primary human epithelial cells. This *ex vivo* cell culture exhibits an appropriate cell polarization for a more physiological-like bacteria-host cell interplay and thus represents a powerful tool for AIEC-infection studies. Throughout the next sections we will detail the entire procedure by which ODMs are obtained and lately infected with AIEC. To that end, we will also specify the performed infection-kinetics assay to determine the ideal time of infection and the bacteria/IEC ratio for this pathobiont to efficiently invade ODMs.

## Materials and Equipment

### Reagents

#### Biological Reagents

- Human Epithelial Organoid 3D Cultures (EpOCs): intestinal samples of healthy sigmoid colon with no evidence of macroscopic inflammatory lesions were obtained from subjects undergoing surgery for left-sided colorectal cancer (CRC) or routine endoscopy for CRC screening. For surgical pieces, a segment of healthy mucosa was collected at least 10 cm from the margin of the affected area. Biopsy samples showed no evidence of neoplastic lesions. However, biopsies were not specifically assessed for signs of microscopic inflammation.
  Surgical or biopsy samples were immediately used for generating EpOCs. **Supplementary Table 1** shows the clinical and demographic characteristics of the subjects enrolled to develop this protocol and from which 3D cultures were obtained. EpOCs samples were used on day 5 of expansion and were distributed among different subgroups based on the experimental approaches used. Patients were recruited at the Department of Gastroenterology, Hospital Clinic Barcelona. The study protocol was approved by the Ethics Committee of the Hospital Clinic of Barcelona (registration number HCB/2016/0546).
- Cell lines: Intestine-407 – I407 – (ATCC CCL-6, RRID: CVCL_1907) cell line.
- Bacterial Strains: The AIEC strain LF82, which was isolated from a chronic ileal lesion of a patient with CD, and the non-pathogenic strain *E. coli* K12 C600 [a prototypical derived laboratory strain which has been extensively used for molecular microbiology and bacterial physiology studies since its isolation in 1954 (43)], were provided in 2006 by Prof. Arlette Darfeuille-Michaud (Université d’Auvergne, Clermont-Ferrand, France).

#### Primary Cell Culture Reagents

All concentrations shown here correspond to the used working concentration (WC).

- Heat inactivated – at 56°C for 30 minutes – fetal bovine serum – FBS – South American (Applied Biosystems, Foster City, CA, USA. Ref. 10270106).
- Washing medium (WM) (**Supplementary Table 2**).
- Matrigel Growth Factor Reduced Basement Membrane (Corning, NY, USA. Ref. 356231): -80°C stored bottles were thawed overnight (ON) on ice. 500 µl aliquots were prepared and frozen at -20°C for later use. Once thawed, aliquots were stored at 4°C for no longer than one week.
- Cell Recovery solution (Corning, NY, USA. Ref. 354253).
- Dissociation medium (**Supplementary Table 3**).
- Wnt3a-conditioned medium + Y (STEM+Y medium) (**Supplementary Table 4**).
- Trypan blue Solution (Gibco, Grand Island, NY, USA. Ref. 15250061).
- Differentiation medium (DIFF medium) (**Supplementary Table 5**).

#### Cell Line Reagents

- Trypsin-EDTA (Lonza, Basel, Switzerland. Ref. H3BE17-161E). WC: 170,000 U/L trypsin and 200mg/L EDTA.
- EMEM Complete Medium (**Supplementary Table 6**).

#### Bacterial Culture Reagents

- Liquid Luria-Bertani (LB) Broth (Sigma-Aldrich, Saint Louis, MO, USA. Ref. L3022).

#### Gentamicin Protection Assay Reagents

- Minimal media (EMEM-MM/DIFF-MM; **Supplementary Tables 7** and **8**, respectively).
- Minimal media containing 100 µg/ml of gentamicin (Lonza, Basel, Switzerland. Ref. 17-519Z).
- Ringer Solution (Scharlau, Barcelona, Spain. Ref. 06-073-500).
- LB Agar (**Supplementary Table 9**).

#### RNA Isolation and Quantitative Multiplex Real-Time Polymerase Chain Reaction Reagents

- TRIzol reagent (Life Technologies, Carlsbad, CA, USA. Ref. 15596018).
- Chloroform (Sigma-Aldrich, Saint Louis, MO, USA. Ref. C2432-500).
- RNeasy Kit (Qiagen, Hilden, Germany. Ref. 74106).
- High Capacity cDNA Reverse Transcription kit (Applied Biosystems, Carlsbad, CA, USA. Ref. 4368813).
- RNAse Inhibitor (Applied Biosystems, Carlsbad, CA, USA. Ref. N8080119).
- TaqMan™ Fast Universal PCR Master Mix (2X), no AmpErase™ UNG (Applied Biosystems, Carlsbad, CA, USA. Ref. 4366073).
- Nuclease Free Water (Promega, Madison, WI, USA. Ref. P1193).
- Pre-designed TaqMan Assays (Applied Biosystems, Carlsbad, CA, USA.): *MYC* (Mm00487804_m1)*, MKI67* (Mm01278617_m1)*, AXIN2* (Hs00610344_m1), *TJP3 (*Hs00274276_m1*), TFF3* (Hs00902278_m1)*, MUC2* (Hs03005094_m1)*, LGR5* (Hs00173664_m1), *FYN* (Hs00176628_m1), *CDCA7* (Hs00230589_m1), *ZG16* (Hs00380609_m1), *TLR3* (Hs01551078_m1), *TLR4* (Hs00152939_m1), *CCL20* (Hs01011368_m1), *CXCL1* (Hs00605382_gH), *CXCL2* (Hs00601975_m1), *ANPEP* (Hs00952642_m1), *FABP2* (Hs01573164_g1), *AQP8* (Hs00154124_m1), *CA1* (Hs01100176_m1), *CHGA* (Hs00154441_m1), *CEACAM7* (Hs03988977_m1), *OCLN* (Hs00170162_m1), *PHGDH* (Hs01106330_m1), *CYP1B1* (Hs00164383_m1), (all of them conjugated with FAM dye) and *ACTB* (endogenous control; Ref. 4310881E) with VIC dye.

#### Immunostaining Assay Reagents

- Paraformaldehyde aqueous solution – PFA – (Electron Microscopy Sciences, Hatfield, PA, USA. Ref. 15710. WC: 4%.
- Glycine (Sigma-Aldrich, Saint Louis, MO, USA Ref. G7126). WC: 20 mM.
- Bovine serum albumin – BSA – (Sigma-Aldrich, Saint Louis, MO, USA Ref. T8787). WC: 1%.
- Primary antibodies: mouse anti-EpCAM (1:100; Dako, Denmark. Ref. M0804), rabbit anti-E-Cadherin (1:100, Cell Signaling Technology, Danvers, MA, USA. Ref. 3195S), mouse anti-KI67 (1:100, Leica, Wetzlar, Germany. Ref. NCL_L-KI67_MM1), rabbit anti-MUC2 (1:250, Santa Cruz Biotechnology, Dallas, TX, USA. Ref. sc-15334), mouse anti-VILLIN (1:100; Dako, Denmark. Ref. M3637) all diluted in 1% BSA.
- Secondary antibodies: anti-mouse Cy3 (1:400, Jackson ImmunoResearch, Cambridge, UK. Ref. 115-165-205. RRID: AB_2338694) and anti-rabbit Alexa 488 (1:400, Jackson ImmunoResearch, Cambridge, UK. Ref. 111-545-144. RRID: AB_2338052) all diluted in 1% BSA.
- 4′,6-diamidino-2-phenylindole (DAPI) (diluted 1:10000 in DPBS, Invitrogen, Carlsbad, CA, USA. Ref. D1306).
- Alexa Fluor™ 555 Phalloidin (diluted 1:40 in 1%BSA; Invitrogen, Carlsbad, CA, USA. Ref. A34055).
- Mounting medium: glycerol (Sigma. Ref. G5516-500). WC: 80%.

#### Other Reagents

- Dulbecco phosphate-buffered saline – DPBS – (Gibco, Grand Island, NY, USA. Ref. 14190-169).
- Triton X-100 (Sigma-Aldrich, Saint Louis, MO, USA. Ref. T8787).
- Distilled H_2_O.
- CellTox™ Green Cytotoxicity Assay (Promega, Madison, WI, USA. Ref. G8741).
- Digitonin (Sigma-Aldrich, Saint Louis, MO, USA., Ref. D141). WC: 100 µg/ml.

### Equipment

#### Consumables

- 1.5 ml tubes (Eppendorf, Hamburg, Germany. Ref. 211-2130).
- 1.5 ml tubes RNAse free (Invitrogen, Carlsbad, CA, USA Ref. AM12400).
- Falcon 15ml Sterile Disposable Conical Centrifuge Tubes (BD Biosciences, San Jose, CA, USA. Ref. 352096).
- Falcon 50ml Sterile Disposable Conical Centrifuge Tubes (BD Biosciences, San Jose, CA, USA. Ref. 352070).
- Filtered pipette tips – 10 µl, 20 µl, 200 µl, 1000 µl – (VWR International Eurolab, Barcelona, Spain. Refs. 732-1148/732-1150/732-1153/732-1154).
- Serological pipettes: 5, 10, 25 ml and 50 ml (VWR International Eurolab, Barcelona, Spain. Refs. 357543/357551/357535/734-1740).
- Scalpels (VWR, International Eurolab, Barcelona, Spain. Ref. SWAN6608).
- Microscope slides (DDBiolab, Barelona, Spain. Ref. 37519).
- KOVA^®^ Glasstic Slide 10 With Counting Grids (Kova, Garden Grove, CA, USA. Ref. 87144E).
- BD Emerald 5 ml syringes (BD Biosciences, San Jose, CA, USA. Ref. 1026307731).
- BD Microlance^®^ 3 21Gx1’’ 0.8mmx25mm (BD Biosciences, San Jose, CA, USA. Ref. 301156).
- MicroAmp™ Optical Adhesive Film (Applied Biosystems, Foster City, CA, USA. Ref. 4311971).

#### Plates and Flasks

- 48-well plates (Corning, NY, USA. Ref. 3548).
- 24-well plates (Jet Biofil, Guangzhou, China. Ref. TCP-011-024).
- µ-Slide 8 Well ibiTreat: #1.5 polymer coverslip, tissue culture treated, sterilized (IBIDI, Gräfelfing, Germany. Ref. 80826).
- T25 and T75 tissue culture flasks (BioLab, Barcelona, Spain. Refs. 55400/55402).
- 120x120mm Petri dishes (Corning, NY, USA. Ref. GOSSBP124-05).
- Microplate 96 well qPCR FAST THERMAL CYCLING (Applied Biosystems, Foster City, CA, USA. Ref. 4346907).

#### Lab Equipment

- Vortex mixer.
- Thermo Scientific™ NanoDrop™ One^C^ Microvolume UV-Vis Spectrophotometer Precision Scale.
- Veriti 96-well Thermal Cycler (Applied Biosystems).
- Benchtop shaker (BOECO Mini-Rocker Shaker MR-1).
- Benchtop refrigerated centrifuge (for 1.5 ml, 15 ml and 50 ml conical tubes).
- Inverted microscope (Olympus X51 Inverted Microscope).
- Fluorescence Inverted Microscope Nikon S Ti.
- Cell incubator (37°C, 5% CO_2_).
- Biosafety hood.
- Autoclave.
- Spectrophotometer.
- ABI PRISM 7500 Fast RT-PCR System (Applied Biosystems).
- Leica TCS_SP5 scanning spectral confocal microscope (Leica Microsystems, Germany) equipped with an DMI 6000 inverted fluorescence microscope, blue diode (405nm), Argon (488nm), diode pumped solid state (561nm) lasers and a Apochromat 63X oil immersion objective (NA 1.4).
- Zeiss LSM880 laser scanning spectral confocal microscope (Carl Zeiss, Jena, Germany) equipped with an Axio Observer 7 inverted microscope, blue diode (405nm), Argon (488nm), diode pumped solid state (561nm) and HeNe (633nm) lasers and a Plan Apochromat 63X oil (NA 1.4) immersion objective lenses.

#### Other Equipment

- Micropipettes and Pipettor.
- Tube racks.
- Refrigerated racks.
- Aluminum foil.
- Cell-counter.
- Forceps.
- Scissors.
- Spectrophotometer Cuvettes.

#### Software Equipment

- Image processing software (Image J Fiji, https://imagej.net/Fiji).
- Data software analysis Graphpad Prism 5 (GraphPad Software, http://www.graphpad.com/).

## Methods

Our prime aim was to develop a new model of infection using primary human intestinal epithelium. For that purpose, ODMs were generated from EpOCs and differentiated (d-ODMs) before being infected by *E. coli*.

In this section we will accurately describe the optimized protocol for ODM generation from EpOCs, ODM differentiation and AIEC infection of d-ODMs to evaluate AIEC’s invasive capacity in differentiated primary epithelial cells.

### Organoid-Derived Monolayer (Timing 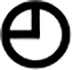 4d)

#### Generation of Organoid-Derived Monolayers

EpOCs were generated as previously described (44, 45). Briefly, crypts were isolated from intestinal samples after an incubation of 45’ with 8mM EDTA at 4°C. Crypts were then embedded in 25 µl of Matrigel and covered with 250 µl of STEM medium (**Supplementary Table 4** – modified without Y). After 2-3 days, the crypt culture was mechanically dissociated to single cells using a dispase-based solution (**Supplementary Table 3**) and expanded at a 1:3 dilution. EpOCs were used after 5 days of expansion to generate ODMs as detailed below. Prior to EpOCs dissociation, 48-well plates were pre-coated with a thin layer of diluted (1:20) Matrigel in DPBS to promote cell adhesion. A volume of 150 µl/well was added and plates were incubated at room temperature (RT) for 1h. Excess Matrigel was discarded and the diluted-Matrigel layer was covered with Advanced DMEM/F12 medium and kept at RT until immediate use. Alternatively, coated plates were stored at 4°C covered in DPBS for up to 7 days.

**Δ CRITICAL.** Based on our experience, every EpOCs drop contains around 40,000-100,000 cells. Thus, depending on the final number of single cells needed for the invasion assay, a determined number of EpOCs drops will be used at the starting point.

**Figure 1:**
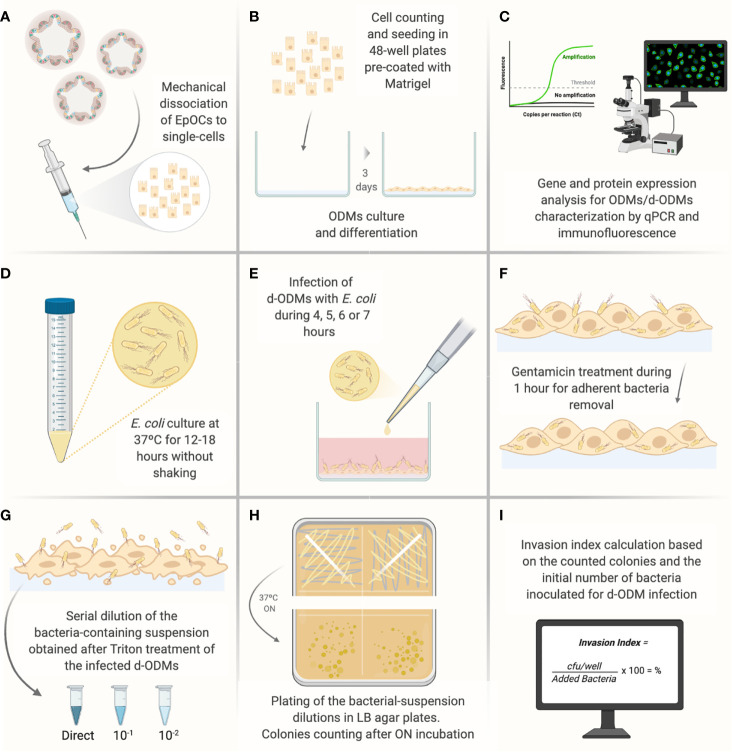
Illustrated experimental workflow of the most critical steps of the d-ODMs-*E. coli* infection protocol. **(A)** Mechanical dissociation of EpOCs with the help of a 5 ml syringe with a 21G needle until achievement of single-cells. **(B)** Single-cell counting and seeding (2x10^5^ cells/well on pre-coated 48-well plates with diluted Matrigel (1:20). Cells were incubated until ODM formation for further differentiation. **(C)** Characterization of ODMs and d-ODMs by qPCR and immunostaining (only during the protocol set-up). **(D)** ON growth of *E*. *coli* LF82 and K12 strains were grown in liquid LB. **(E)** Infection of d-ODMs with *E*. *coli* strains performed by gently releasing the drop. Infection times were from 4-7 hours. **(F)** Gentamicin (100 µg/ml) addition for 1 hour to eliminate adherent bacteria. **(G)** Cell treatment with 1% Triton X-100 to facilitate intracellular bacteria release. The bacterial suspension was then serially diluted and seeded. **(H)** ON incubation of bacterial dilutions in LB agar plates. **(I)** After colony counting, the Invasion Index for each strain was determined. This figure was created using BioRender.com.

To generate ODMs from EpOCs, the protocol was as follows:

(1) Matrigel drops containing EpOCs were washed with cold DPBS and collected in Cell Recovery solution (300 µl/well) at 4°C for 40 minutes. Every 5-10 minutes, cell suspensions were gently inverted upside-down.
(2) 4-5 ml of washing medium (WM) (**Supplementary Table 2**) were added, and the cell suspensions were centrifuged at 400*g* for 4 minutes at 4°C.
(3) Supernatant was discarded and the pellet was resuspended in Dissociation Medium (**Supplementary Table 3**) followed by 15-20 minutes of incubation at 37°C. On average, 5 ml Dissociation Medium were used for every 20-25 Matrigel drops.
(4) After organoid release, cells were mechanically disaggregated using a 5 ml syringe with a 21G needle until the cells were totally dissociated (20-50 strokes were conducted depending on the sample (**Figure 1A**)). To evaluate the extent to which EpOCs were dissociated to single cells, microscope observation was performed. If required, additional rounds of 10-20 strokes followed by microscope observation were performed until complete cell dissociation was reached.
(5) Cells were centrifuged at 800*g* at 4°C for 4 minutes and washed with 5 ml of WM after supernatant removal. This step was repeated twice.
(6) The remaining pellet was resuspended in 1-2 ml of WM for manual cell counting:
a. Cells (10 µl) were diluted 1:1 with Trypan blue Solution.
b. 10 µl of the cell suspension was loaded into a Glasstic Slide 10 With Counting Grids and the cell number was estimated according to the manufacturer’s recommendations. The mortality rate (% of dead cells over the total number of cells) was usually below 10%.
(7) Single cells were again centrifuged, and the pellet was resuspended in the required volume of STEM+Y medium (**Supplementary Table 4**) to achieve 2x10^5^ cells/well/250 µl.
(8) Cells were seeded on Matrigel pre-coated 48-well plates and incubated for 24h at 37°C 5% CO_2_ (**Figure 1B**).

#### Differentiation of Organoid-Derived Monolayers

After incubation, ODMs were induced to differentiation. To this end, STEM + Y medium was discarded and ODMs were washed with DPBS and Advanced DMEM/F12 medium (300 µl/well) at RT to remove dead cells. DIFF medium (250 µl/well) (**Supplementary Table 5**) was then added and ODMs were incubated at 37°C 5% CO_2_ for an additional 48h.

**Figure 2:**
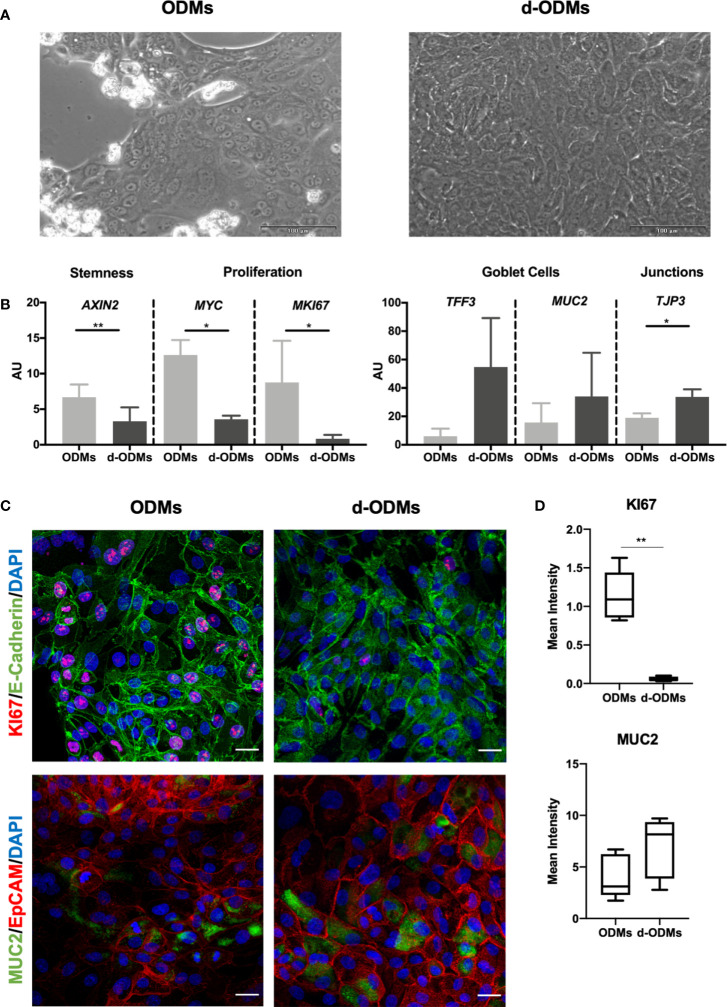
Organoid-Derived Monolayers (ODMs) characterization. **(A)** ODMs (left panel) 24 hours after seeding showed a confluence of around 70-80% and d-ODMs (right panel) 48 hours after differentiation, showed 100% confluence. **(B)** Gene expression analysis of ODMs and d-ODMs (n = 5 for each culture type). *AXIN2, MYC, MKI67, TFF3*, *MUC2* and *TJP3* genes were analyzed by qPCR to determine their expression levels in ODM vs. d-ODMs. A paired t-test was performed to examine statistically different expression patterns between the two groups (ODMs/d-ODMs). A P value of <0.05 was considered statistically significant. *AXIN2*: ** indicates P = 0.0012. *MYC*: *indicates P = 0.0135. *MKI67*: *indicates P = 0.0335. *TJP3*: *indicates P = 0.0365. **(C)** Protein expression analysis by immunofluorescence. KI67 and MUC2 were analyzed to confirm the proliferation and differentiation status of both ODMs and d-ODMs. E-Cadherin and EpCAM were used as epithelial cell-wall markers. DAPI was used to counterstain the cell nuclei. Scale bars: 25 µm. Images are representative of n = 3 independent experiments performed with samples from two different donors. **(D)** Box-plot distribution of the fluorescent signal of KI67 and MUC2 proteins in ODMs and d-ODMs, expressed as Mean Intensity. Fluorescence was quantified in 5 different fields per sample. A paired t-test was performed to examine statistically different expression patterns between the two groups (ODMs/d-ODMs). A P value of <0.05 was considered statistically significant. KI67: **indicates P = 0.0013.

Under these conditions, the differentiated monolayer (d-ODMs) reached 100% confluence 1-2 days after differentiation (**Figure 2A**). Therefore, the period between cells seeding and infection was 72h (cells were incubated for 24h after seeding and before differentiation, and 48h after differentiation and before infection).

#### Quantitative Multiplex Real-Time Polymerase Chain Reaction and Immunofluorescence

**Δ FOR SYSTEM SET UP ONLY.** The methodology described in this section was only utilized during optimization and until the protocol we established was entrenched (**Figure 1C**).

##### RT-qPCR

Both ODMs and d-ODMs were harvested in Trizol for RNA extraction (**Supplementary Table 1 Group 1**) and isolation using the RNeasy Kit. RNA was transcribed to cDNA at a final concentration of 250 ng/50 µl using the reverse transcriptase High-Capacity cDNA RT kit with RNase inhibitor. Reverse transcription was performed using a Programmable Thermal Cycler for 10 minutes at 25°C followed by 2 hours at 37°C. Quantitative Multiplex Real-time PCR (qPCR) was then conducted to characterize the monolayer gene expression pattern in ODMs versus d-ODMs. qPCR 96-well microplates contained a .volume of 10 µl/well (1 µl cDNA+0.5 µl each TaqMan Assay diluted in TaqMan Fast Universal PCR Master Mix and H_2_O). Target genes were amplified and quantified using *ACTB* as the endogenous control. PCR reaction was run in the ABI PRISM 7500 Fast RT-PCR System using the following program: a holding stage for 20 seconds at 95°C and a cycling stage for 3 minutes at 95°C and 30 seconds at 60°C during 40 cycles. Target gene expression values relative to *ACTB* were expressed as arbitrary units (AU) following this formula:

$$AU=\;2^{-(\mathrm{Ct}\;\text{target gene}\;-\;Ct\;ACTB)\;}\times 1000$$

##### Immunofluorescence Staining

Monolayer cultures (both ODMs and d-ODMs, **Supplementary Table 1 Group 3**) seeded in µ-Slide 8 Well ibiTreat chambers (for optimal image acquisition) were processed for immunofluorescent staining as follows:

(1) After two DPBS washes, the cell monolayer was fixed with 2% PFA (1:1 4% PFA + DPBS) for 5 minutes at RT and then with 4% PFA for 10 minutes at RT.
(2) Cells were washed three times with DPBS: 1^st^ fast; 2^nd^ and 3^rd^ 5 minutes at RT.
**Δ CRITICAL**. STOP POINT – Cells were stored at 4°C covered in DPBS (300 µl) or were immediately used for staining.
(3) 250 µl of 20mM Glycine was added for 10 minutes at RT to reduce background staining.
(4) DPBS washes were conducted as described in step (2).
(5) For permeabilization, 250 µl of 0.25% Triton X100 were added for 20 minutes at RT.
(6) Cells were then washed 3 additional times – 5 minutes each – with DPBS.
(7) To block non-specific binding, 250 µl of 1% BSA was applied and incubated at RT for 30-45 minutes.
(8) Primary antibodies (150-200 µl/well) – EpCAM, E-Cadherin, MUC2, Villin or KI67 – were added at the specified dilutions (in 1% BSA) and incubated ON at 4°C.
(9) After 3 DPBS washes (as in step 6), cells were incubated with 150-200 µl/well of the secondary antibodies – Anti-mouse Cy3 and Anti-rabbit 488 – at the specified dilutions (see *Materials* section) in 1% BSA for 1h of incubation at RT. Cells were washed 3x with DPBS at RT as described in step 6.
(10) For DNA counterstaining, DAPI (250 µl/well) was added and incubated at RT for 10 minutes. Washes were repeated as in step 6.
(11) Finally, 200 µl/well of mounting medium (80% Glycerol in DPBS) were added. Samples were stored at 4°C for subsequent fluorescent microscope observation.

**Δ CRITICAL**. After adding the secondary antibodies, cells were kept in the dark.

**Δ CRITICAL**. For short-term storage, stained cells were kept at 4°C or at -20°C for up to 6 months.

### AIEC Infection of Differentiated Organoid-Derived Monolayer (Timing 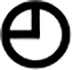 3d)

#### Bacterial Strains

Prior to infection, LF82 and *E. coli* K12 strains were cultured in 1.5 ml of LB Broth and incubated for 12-18 hours at 37°C without shaking (**Figure 1D**).

#### Reference Model of Infection

The I407 cell line, originally employed for AIEC-pathotype identification (6), was used as the reference method of the gentamicin protection assay in order to ensure that the bacterial strains ON cultures show the expected phenotype. Cells were passaged every 2-3 days *via* 5-minute incubation with 1 ml of Trypsin-EDTA after a washing step with DPBS. After collection, cells were centrifuged at 500*g* for 5 minutes and 20°C. Pelleted cells were resuspended in EMEM complete medium (**Supplementary Table 6**) and seeded in T75 flasks. Twenty-four hours before infection, 4x10^5^cells/well were plated on 24-well plates.

The assay was performed at Multiplicity of Infection (MOI) 10, as described previously (46, 47). Infection lasted 3 hours followed by 1 hour of gentamycin treatment. During the entire procedure, EMEM-MM (**Supplementary Table 7**) was employed. Invasive ability was quantified as the percentage of the intracellular bacteria from the initial inoculum (4×10^6^ cfu/ml):

$$\text{I-INV}\;(\%)=\left(\text{intracell. bacteria}/4\times 10^{6}\right)\times 100$$

**Δ FOR SYSTEM SET UP ONLY.** This model of infection was only performed until establishment of the d-ODM-based gentamicin protection assay.

#### d-ODM-Based Gentamicin Protection Assay

##### d-ODM Cell Counting

To infect cells with a determined MOI, it is crucial to know the exact number of cells seeded as a monolayer at the time of infection. In our particular case, we seeded 2x10^5^ EpOCs-derived single cells/well in 48-well plates based on previous experience (data not published), although this may need to be adjusted by each lab as culture conditions can vary slightly. To monitor the number of cells present in the plate at 100% confluency, experiments were performed seeding the above number of cells/well and counting cells present in d-ODM prior to infection. This step proved decisive in order to adjust the needed inoculum of bacteria and achieve the desired MOI. Briefly, d-ODMs were washed with DPBS to remove non-attached cells. Trypsin-EDTA (150 µl) was added to the culture for 10-15 minutes at 37°C 5% CO_2_. Detached cells were collected and resuspended in Advanced DMEM/F12 + 10% FBS. These last two steps were repeated until complete cell-detachment was achieved. Cells were centrifuged at 800*g* for 4min and at 4°C and resuspended in 200 µl of Advanced DMEM/F12 + 10% FBS for cell counting as explained in a previous section (see the *ODM generation* section).

**Δ CRITICAL.** It is important to not exceed the 10-15 minutes incubation with Trypsin-EDTA in order to prevent cell death.

On average, we recovered approximately 1.8x10^5^ cells/well prior to infection (**Supplementary Figure 1**), which is close to the number of cells initially seeded. Notice that these numbers may have to be adjusted by each lab, as mentioned above.

For the infection assay, two different MOI – 20 and 100 – were assessed on d-ODM-based assays.

Thus, d-ODM counted-cells (1.8x10^5^ cells/well) were multiplied 20- or 100-times to determine the bacterial colony forming units (cfu)/ml required for reaching each MOI value. In our case, 3,6x10^6^ or 18 x10^6^ *E. coli* cfu/ml were needed.

**Δ CRITICAL.** Working at a confluence as close as possible to 100%, is essential to ensure the optimal ratio of bacterial cells/eukaryotic cells in order to reach the desired MOI.

##### Bacterial Optical Density and Colony Forming Unit Adjustment

The study of the *E. coli* growth curve in LB allowed us to estimate the cfu/ml at every measured Optical Density (OD) (**Supplementary Figure 2**). Prior to infection, ON bacterial cultures (both from LF82 and K12 strains) were adjusted to OD = 0.1, corresponding to 1.6x10^8^ cfu/ml. This OD was chosen since it represents an adequate inoculum volume for the infection assay for both of the assessed MOIs. The bacterial suspension was prepared following these steps:

(1) ON bacterial cell suspensions (500 µl) were diluted 1:1 with LB medium and 1 ml was transferred to a cuvette.

(2) The OD was measured with a spectrophotometer at a wavelength (λ) of 600 nm.

(3) OD adjustment was achieved in accordance with the following formula:

iV=fOD (0.1)×fV/(mOD)× 2

iV; Initial Volume (required volume for the ON culture)

fOD; Final OD (0.1 in this case)

fV; Final Volume (1 ml)

mOD; Measured OD

(4) The calculated iV and DIFF-MM (**Supplementary Table 8**) up to 1 ml total volume were added to a 1.5 ml tube.

##### ODM Infection and Gentamicin Protection Assay

As already mentioned, LF82 and K12 strains were used as positive (invasive) and negative (non-invasive) control, respectively. Infection was performed using d-ODMs generated from 7 different subjects (**Supplementary Table 1 Group 2**) as the starting material. Every experiment was conducted in duplicate.

DIFF medium was discarded from 100% confluent d-ODMs; cells were washed twice with DPBS at RT (500 µl/well) and fresh DIFF-MM was added (500 µl/well). Then, the corresponding volume of OD 0.1 bacterial suspension (**Table 1**) was inoculated to reach each assessed MOI by gently releasing the drop (**Figure 1E**). Infected d-ODMs were incubated for 4, 5, 6 or 7 hours at 37°C 5% CO_2_ for the complete infection-kinetics study. At the end of each time point, cells were washed 3 times with DPBS at RT – as explained above – and DIFF-MM containing 100 µg/ml of gentamicin was added for 1 additional hour (**Figure 1F**) in order to remove the extracellular bacterial cells. Three more DPBS washes at RT were required after gentamicin treatment. 1% Triton X-100 (250 µl/well) was added to d-ODMs to release the internalized bacteria. Vigorous pipetting to generate bubbles was required to efficiently detach and break the eukaryotic cell membranes (**Figure 1G**).

**Table 1:** Adjustment of the added bacterial-culture volume to the d-ODM culture depending on the tested MOI.

MOI 20	MOI 100
Number of d-ODM-cells: 180,000	Number of d-ODM-cells: 180,000
Final cfu/ml (fC): 3,600,000	Final cfu/ml (fC): 18,000,000
Final volume/well (fV): 500 µl	Final volume/well (fV): 500 µl
Initial cfu/ml (iC): 1.6x10^8^	Initial cfu/ml (iC): 1.6x10^8^
Added volume (_add_V): **11.25 μl**	Added volume (_add_V): **56.25 μl**

**Δ CRITICAL**. The Triton X-100 step should not take longer than 30 minutes in order to avoid bacterial cell death.

##### Invasion Index

To be able to count cfu/ml, the bacterial suspension resulting from the Triton X-100 treatment was serially diluted in Ringer Solution (**Figure 1G**). Dilutions of 10^-1^ and 10^-2^, as well as the non-diluted samples, were plated (25 µl) in LB agar plates (**Supplementary Table 9**) and incubated ON at 37°C.

**♦ TIP**. 120x120mm square plates were used to plate up to 4 different dilutions. Plating was performed with the pipette-tip itself immediately after inoculation. The inoculum was streaked homogeneously through the plate-section (**Figure 1H**).

**Δ CRITICAL**. For a homogeneous mixture of bacterial dilutions, vortexing solutions is highly recommended.

Grown colonies in each dilution were only taken into consideration when the counting was between 15 - 150 (**Figure 1I**).

$$Intracellular\;bacteria=\;\frac{\Sigma \;colonies}{\left(0.025\;\times \;\left(n_{1}+0.1\;x\;n_{2}\right)\;\times \;DF\right)}\;\times \;well\;volume\;(0.25)=cfu/well$$

n_1_ = number of plates at the more concentrated dilution

n_2_ = number of plates at the less concentrated dilution

DF = dilution factor of the more concentrated dilution

Once the number of cfu/well was obtained, the invasion index (%) was calculated considering the amount of bacteria initially inoculated to d-ODMs:

$$Invasion\;Index=\;\frac{Intracellular\;bacteria}{Inoculated\;Bacteria†}\;\times \;100=\%$$

†: in this context, 3.6x10^6^ for MOI 20 or 18 x10^6^ for MOI 100.

As previously described by Darfeuille-Michaud et al., who studied AIEC infection by using immortalized cell lines (6), we considered a strain to be invasive when the Invasion Index was > 0.1%

#### Fluorescent Cyto-staining and CellTox Green Cytotoxicity Assay

Notice that even though the techniques detailed herein are not mandatory, they were performed to obtain a deeper understanding of the results obtained from the AIEC infection of d-ODM (see *Anticipated Results* section).

##### Fluorescent Cyto-Staining

To visualize the bacterial internalization, LF82- and K12-infected monolayer cultures (at 5 hours of infection followed by 1 hour of gentamicin treatment (5 + 1) and MOI 100) seeded in µ-Slide 8 Well ibiTreat chambers, were processed for fluorescent cyto-staining. This procedure was identical to that used for ODM/d-ODM characterization until step (7) of the Immunofluorescence Staining section. After incubation with the blocking solution, 150-200 µl/well of Phalloidin diluted 1:40 in 1% BSA was added for staining of the actin filaments. After 1-hour incubation at RT, cells were washed 3x with DPBS at RT as in step 6 (see *Immunofluorescence Staining* section). DAPI (250 µl/well) was then added and the protocol continued as described in steps 10 and 11. The assay was performed with cells obtained from 3 different subjects (**Supplementary Table 1 Group 3**).

##### CellTox Green Cytotoxicity Assay

The protocol for d-ODMs cytotoxicity assessment corresponded to that recommended by the manufacturer. Briefly, after the infection assay, infected and non-infected d-ODMs were incubated with the CellTox reagent (1:1, 150 µl DIFF-MM + 150 µl CellTox) previously diluted according to the manufacturer’s instructions (1:500 in Assay Buffer). After ≥15 minutes of incubation at 37°C in the dark, cultures were observed using a fluorescence microscope. A positive control of cell death was included by adding 100 µg/ml of digitonin in the uninfected d-ODM for 1 hour.

### Data and Statistical Analysis

Quantitative data are expressed as the standard error of the mean (SEM). A paired t-test was performed to examine statistically different expression patterns between 2 groups, and a 2-way ANOVA test to examine statistical significance in multiple group data sets, followed by a Tukey test correction for multiple testing. A p-value of <0.05 was considered statistically significant. Data were analyzed using Graphpad Prism 8 (version 8.2.1).

## Anticipated Results

### Establishment of Differentiated Human Intestinal Epithelial Monolayer Cultures

The intestinal crypt is organized so that the stem-cell compartment resides at the bottom, thereby protected from the luminal content, while the differentiated and surface epithelium is more directly in contact with the microbiota and its metabolites. In order to develop a model that would more closely resemble the type of upper crypt epithelium that is more susceptible to bacterial interactions and based on previous results from our lab (48, 50), we used a monolayer of differentiated epithelial cells derived from epithelial organoid cultures (d-ODMs).

First, we aimed to determine the optimal culture conditions for the ODMs to acquire a differentiated phenotype while reaching an appropriate confluence (100%) for the AIEC invasion assay. Based on previous experiments by our lab, we seeded 2x10^5^ single cells/well. On day 1, cells created clusters that alternated with empty areas, while on day 3, the monolayers reached 100% confluence, the requirement for AIEC infection (**Figure 2A**). Under these conditions, cells were collected and counted, obtaining an average of approximately 1.8x10^5^ cells/well (**Supplementary Figure 1**). Once the d-ODM number of cells at ~100% confluency was determined, we confirmed the differentiated phenotype of the monolayer by measuring key genes and proteins whose expression changes dramatically upon epithelial stem cell differentiation (38, 50).

As shown in **Figure 2B**, mRNA levels of *AXIN2*, *MYC* and *MKI67*, (the first, marker of stemness and the two last, markers of proliferation), were significantly higher in ODMs compared to d-ODMs. On the other hand, transcriptional levels of the differentiation markers *TFF3* and *MUC2*, showed an up-regulation, despite not statistically significant, in d-ODMs compared to ODMs. Similarly, *TJP3*, representative marker of epithelial cell junctions, was significantly up-regulated in d-ODM. Other markers included in the analysis (**Supplementary Figure 3**) confirmed the differentiated phenotype of the d-ODM culture (48).

Although using transcriptional analysis to easily screen cultures for their differentiation status – or other phenotypic features – is valuable, protein staining of the intact 2D cultures would help evaluate not just protein expression but also localization within the cell monolayer.

As an example, here we determined the protein expression of KI67, MUC2 and Villin by immunofluorescence. **Figure 2C** and **Supplementary Figure 4A** show representative images from 3 independent experiments. In agreement with the differentiated phenotype achieved in d-ODMs, KI67 was markedly decreased while MUC2 and Villin were increased compared to ODMs. These results were confirmed by fluorescence quantification analysis (**Figure 2D** and **Supplementary Figure 4B**).

Finally, to prove that the 2D culture exhibited an appropriate cell polarization, orthogonal views of MUC2 and Villin were analyzed (**Supplementary Figure 5**), showing a marked up-regulation of these two differentiation markers at the apical side of the d-ODM.

Altogether, both approaches demonstrated that primary cells derived from human EpOCs can establish a stable monolayer that preserves the intestinal identity thus mimicking the tissue of origin. Moreover, we achieved a differentiated and polarized phenotype in the d-ODMs at optimal confluence for the AIEC-infection study.

### AIECs Can Invade d-ODMs

**Figure 3:**
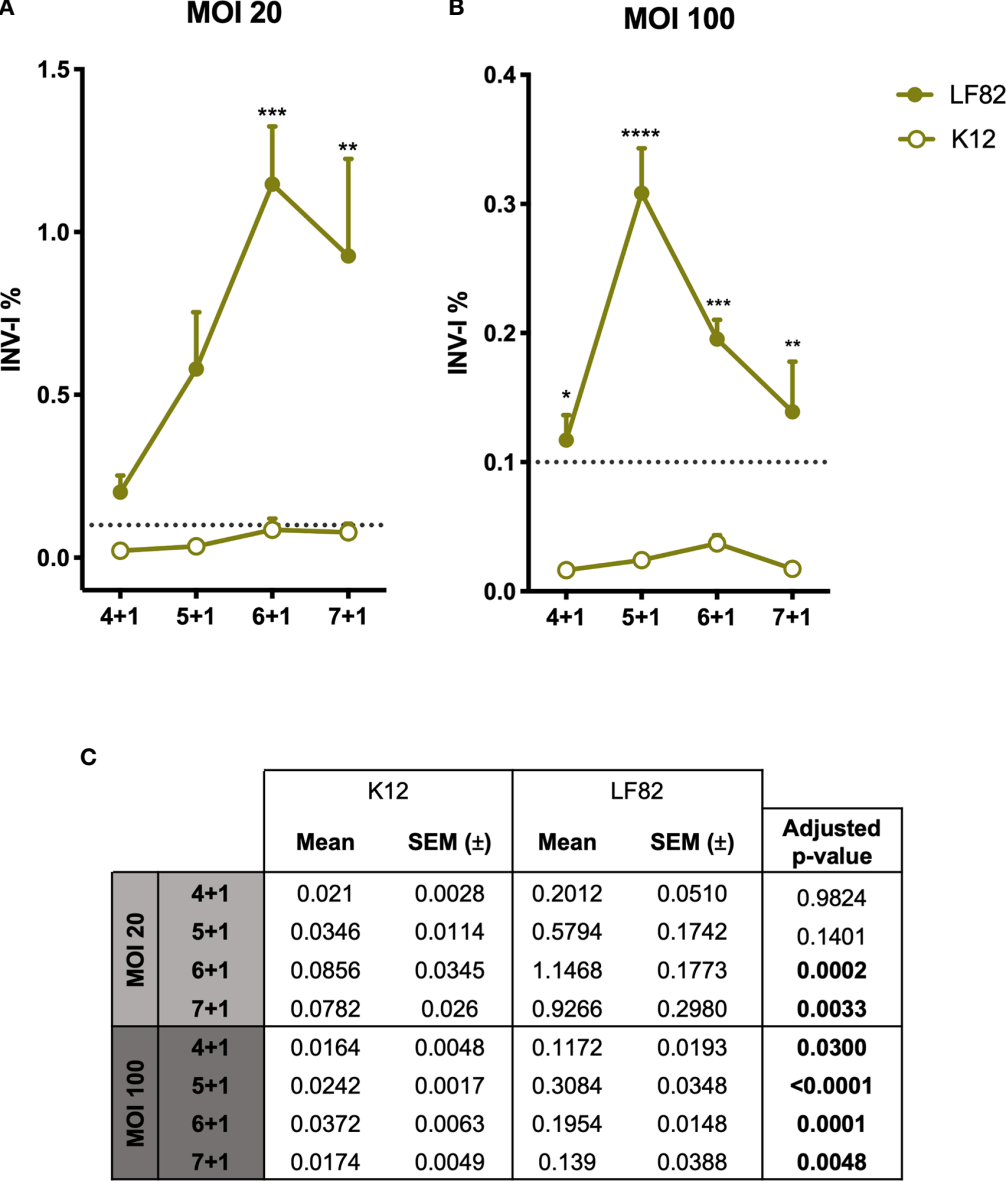
Graphic representation of *E*. *coli* LF82 and K12 invasion indexes on d-ODMs. INV-I% of both *E*. *coli* strains (n = 5 for each represented point in the graph) at MOI 20 **(A)** and 100 **(B)** relative to the increasing infection time points. The dashed line represents the established threshold (0,1) over which *E*. *coli* strains were considered to be invasive. The error bars correspond to the SEM. **(C)** Mean, SEM and adjusted p-values obtained by a 2-way ANOVA test to examine statistical significance between LF82 and K12 INV-I% for each infection timepoint. This analysis was followed by a Tukey test correction for multiple testing. A P value of <0.05 was considered statistically significant, and it is highlighted in bold.

To the date, the characteristics and pathogenicity of AIECs have been studied so far by employing immortalized cell lines (36). Here, we studied the capability of AIECs to interact and invade a primary intestinal monolayer culture. First, we designed a kinetics infection assay to determine the time course of bacterial entry and/or intracellular survival in our culture system. To verify the strains’ invasiveness capacity, I407 cell line was used as the reference method of the gentamicin protection assay. Both invasion assays (d-ODM and I407 infection) were carried out in parallel; thus, the *E. coli* ON cultures used for their infection were the same for each experiment performed. Results represented in **Supplementary Figure 6** show an INV-I% in I407 cells of 0.99 ± 0.225 and 0.0025 ± 0.00094 for the LF82 and K12 strains, respectively. These results were in agreement with previously published data (6, 46). Therefore, we conducted an infection-kinetics study to examine AIEC-d-ODMs infection by determining the percentage of internalized bacteria every hour for 7 hours of infection followed by 1 hour of gentamicin treatment as detailed in the previous sections. The assessed MOIs were 20 and 100. As shown in **Figures 3A, B**, we could quantitatively prove that the AIEC LF82 strain was able to invade d-ODMs, while the non-invasive *E. coli* strain (K12) showed an invasion index (INV-I%) below the established background (<0.1%). Moreover, LF82 showed a time-dependent increment of the INV-I%, and thus of the invasion capacity and/or intracellular multiplication in the AIEC-reference strain. Nevertheless, this capability was significantly higher compared to the K12 strain, both at 6 and 7 hours after infection for MOI 20 (**Figure 3A**) and at all time points for MOI 100 (**Figure 3B**). In fact, 5 hours of infection followed by 1 hour of gentamicin treatment at MOI 100 showed the greatest difference; the LF82 INV-I% measured almost 13 times greater than the K12 INV-I%. This occurred despite the fact that all INV-I% were lower at MOI 100 than at MOI 20. Furthermore, working with a greater number of bacteria/cell (higher MOI) ensured a remarkable reproducibility over time with highly consistent numbers of internalized bacteria in every experiment performed (**Figure 3C**). Nevertheless, this does not ensure higher INV-I%; in fact, this proved to be higher when the MOI was lower, as shown in **Figures 3A, B**. Maintenance of the d-ODMs cells’ viability throughout all of the timepoints was observed *via* the CellTox Green assay (data not shown).

**Figure 4:**
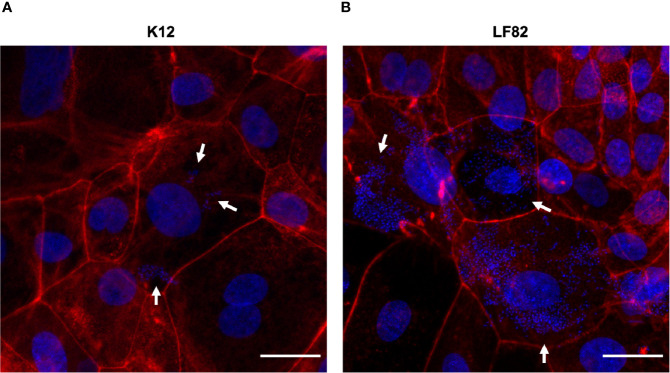
*E. coli* LF82 and K12 invasion of d-ODMs as determined by the gentamicin protection assay. Phalloidin staining was performed to visualize the non-invasive control strain K12 **(A)** and the invasive LF82 **(B)** in d-ODMs after 5 hours of infection and 1 hour of gentamicin treatment at MOI 100. Phalloidin marked the eukaryotic actin filaments while DAPI bound to the DNA of both epithelial and bacterial cells. White arrows show bacterial localization inside the IECs. Scale bars: 25 µm. Images are representative of n = 3 independent experiments performed with samples from two different donors.

By staining the eukaryotic actin filaments (**Figure 4**), we confirmed the presence of high amounts of intracellular LF82 bacteria in the majority of those cells that formed the d-ODMs compared to the K12 strain.

In summary, we demonstrated the capacity of AIECs to invade the epithelial cells of d-ODMs. Thus, we present here a method that can be applied in multiple AIEC-IEC cross-talk studies, not only to discover new AIEC pathogenic mechanisms and host implicated molecules, but also, and more relevantly, to establish a possible starting point for further clinically oriented applications.

## Advantages and Disadvantages

In the following section we will highlight which, in our opinion, are the most noteworthy advantages and disadvantages that this protocol presents. By doing so, we can focus on its practicality and try to overcome its limitations.

### Advantages

- Working with samples isolated from their natural surroundings (the human intestine in this case), preserves the cytoarchitecture and most of the intercellular connections and interactions. Moreover, it also provides the option to consider the interindividual variability that exists between different subjects.
- Working with ODMs and d-ODMs offers accessibility to the IECs-apical side, contrary to 3D-organoid structures which may be required for infectious models.
- We also demonstrate its great reproducibility, a highly relevant feature when one considers the differences between individuals and their responses to microbes.
- Given the fact that ODMs and d-ODMs can be generated from potentially any individual, including patients suffering from IBD, this method offers the possibility of testing personalized treatment approaches.

### Disadvantages

- Time-consuming. EpOCs and ODM cultures are time intensive. Nonetheless, once the system is set up, organoid-derived single cells can be more rapidly obtained, shortening the time required for the entire procedure.
- Costly. EpOCs and ODM cultures could remain unaffordable for some research groups due to the high costliness of most of the reagents that are required.
- Access to patient specimens is required.
- Sample-to-sample variability might lead to differences in the number of cells obtained from every EpOCs drop. This might be an important limiting aspect that should be considered when applying the method described here.

## Discussion

In this manuscript we describe the steps required to develop a novel and reproducible human intestinal epithelial model for the study of enteric bacterial infections, particularly AIEC-related infections. Our model takes into consideration the variability of human biological responses to any pathogens, something that other models based on the use of cell lines cannot fully address (41, 51). Indeed, one of the main advantages of working with *ex vivo* primary cultures (as we mentioned in the previous section) is that these might offer a more physiological view of the host’s response to AIEC infections. However, unpredictable biological variability could hinder the obtainment of the necessary cell concentration at the starting point. In that context, establishing an accurate and standardized protocol is crucial to facilitating reproducibility and enabling results comparisons. In our case, reproducibility was assessed first, by testing the gene and protein expression levels of the 2D cultures derived from the different donors. Moreover, AIEC infections were carried out in duplicate, exposing those EpOCs-derived d-ODMs from seven different individuals to *E. coli*. This validation approach is of great importance in host-pathogen interaction studies, considering the real differences in infection susceptibility among individuals and the divergence in host responses to a pathogen (39).

While a more extensive characterization of the d-ODM at protein level would add robustness to our culture system, our results suggest that ODMs and d-ODMs preserve the characteristics of the intestinal epithelium *in vivo*, resembling cells at the base and top of colonic crypts, respectively. Determining the number of cells that form the monolayer at the time of infection is a crucial step to better adjusting the working conditions in order to (1) achieve the optimal differentiated phenotype of the monolayer cultures, and (2) to properly adjust the number of exposed bacterial cells to the d-ODMs (MOI), which can greatly affect the results.

AIEC infection of d-ODMs was performed at different time points to analyze and select the best condition for achieving high reproducibility of infection and maximum specificity (lowest infection by non-invasive *E. coli*). Over time, increasing amounts of invasive bacteria were detected, with higher values evident when smaller amounts of bacteria (MOI 20) were added to the culture at the starting point. Based on this finding, we concluded that adding more bacteria does not directly correlate with higher invasion values. Similar results were obtained by Boudeau et al. in 1999 with Hep-2 cells (46). A 5-fold increase in the inoculum only represented an increase of 2.06 ± 0.7–fold (mean value of the fold-change increase for each timepoint) in intracellular bacteria. As d-ODMs cells were verified as viable with the CellTox Green assay, differences in the invasion indices were related to the initial inoculum. We believe that the d-ODMs can harbor a limited number of intracellular bacteria and, therefore, upon a given quantity of initial inoculum the invasion index will be lower. Even so, working with higher bacterial loads ensures a remarkable reproducibility of the results. This observation is not only valid for the invasive LF82 strain but also for the non-invasive control, K12.

Another observation concerns the dramatic decrease in the invasion index at the longest time of infection on LF82 INV-I% for both MOI 20 and 100. Other authors have similarly reported a decrease in the intracellular bacteria 4 hours after infection in mouse embryonic fibroblasts and HeLa, Hep-2 and I407 cell lines (53). Initially, we hypothesized that this event might be a consequence of eukaryotic cell death due to the bacterial infection process. Based to this assumption, when the initial bacterial load was higher (MOI 100), eukaryotic cells would have begun dying at earlier time points. Nonetheless, using the CellTox Green assay we observed that infected cells viability was maintained over time (data not shown). Although AIECs are capable of evading IECs and macrophage-related stress responses in order to eliminate intracellular pathogens (6, 7, 46), decreases in the intracellular bacteria could reflect the capacity of IECs to restrict AIEC replication after a certain infection period (52). Testing the intracellular-bacteria viability at each time point would help confirm our hypothesis. It would also be interesting to determine, using this model, the presence of intracellular AIEC cells with a persistent phenotype; i.e. viable bacteria in a non-replicating state (53).

Similar strategies have been applied to study the interaction between AIEC, or other enteric pathogens and *E. coli* pathotypes, and human isolated IECs (23, 37–39, 41) and there is a recent and relevant publication in which organoid-derived 2D cultures are infected with AIEC (42). Nonetheless, this report does not include a detailed description of the steps taken to optimize infection efficacy. In contrast, our focus was to describe the steps required to obtain optimal ODM from EpOCs, that can be used as a model of primary epithelial cell infection with different *E. coli* strains. In particular, we go over the optimized steps from cell counting prior to infection to ODMs differentiation, and from infection kinetics to MOI testing. To the best of our knowledge, this is the first publicly accessible protocol that demonstrates the capacity of AIEC, compared to a non-invasive strain, to infect human primary IECs in a 2D configuration. Nonetheless, in our study we did not evaluate the impact of AIEC infection on epithelial cells including expression of bacterial sensing molecules, tight junctions, or immune response secreted proteins (54, 55). Such studies deserve further attention and will help elucidate how the epithelium differentially responds to invasive compared to non-invasive *E. coli*.

In conclusion, we can report the successful development of a human primary organoid-derived epithelial monolayer model of infection. Further application of this model, such as growing the d-ODMs in transwell-chambers in order to co-culture monolayers with AIECs and other human intestinal cell types (56) or the generation of d-ODMs derived from IBD patients, might lead not only to the development of a more comprehensive approach for studying the interaction of AIECs with the human gut, but also to a better understanding of the pathophysiology underlying inflammatory intestinal disorders.

## Data Availability Statement

The original contributions presented in the study are included in the article/**Supplementary Material**. Further inquiries can be directed to the corresponding author.

## Ethics Statement

The studies involving human participants were reviewed and approved by Ethic Committee of the Hospital Clinic of Barcelona with the registration number HCB/2016/0546. The patients/participants provided their written informed consent to participate in this study.

## Author Contributions

AM, ID, AS, and MM-M designed the study. AM designed and conducted the experiments, acquired and analyzed the data, performed the biostatistics analysis, and wrote the manuscript. ID, designed and conducted experiments. MM-P and ME collected samples and provided technical support. QB-R provided technical support. ER recruited patients and collected samples. ID, AS, and MM-M supervised the experiments, analyzed data, and reviewed the manuscript. All authors contributed to the article and approved the submitted version.

## Funding

AM was supported by the Programa Estatal de Investigación, Desarrollo e Innovación del Ministerio de Economía, Industria y Competitividad, co-funded by the European Social Fund (ESF) under grant agreement number FPI BES-2016-076642. ID is funded by the Horizon 2020 Framework Programme (EU for Research and Innovation H2020), Grant 720905. QB-R was supported by the Programa d’Ajuts per a Investigadors en Formació de la Universitat de Girona – IFUdG 2019/21. AS is funded by the Centro de Investigación Biomédica en Red de Enfermedades Hepáticas y Digestivas (CIBEREHD) and Grant RTI2018- 096946-B-I00 from the Ministerio de Ciencia, Innovación y Universidades, Spain. MM-M is funded by the Spanish Ministry of Economy and Competitiveness through project SAF2017-82261-P, which is co-funded by the European Regional Development Fund.

## Conflict of Interest

The authors declare that the research was conducted in the absence of any commercial or financial relationships that could be construed as a potential conflict of interest.
