# Supplementary material for: A Novel Strategy to Study the Invasive Capability of Adherent-Invasive Escherichia coli by Using Human Primary Organoid-Derived Epithelial Monolayers
Source: Front Immunol. 2021 Mar 29;12:646906. doi: 10.3389/fimmu.2021.646906 (PMC8039293; doi:10.3389/fimmu.2021.646906)
Supplement: Supplementary file 1 [file DataSheet_1.docx]

***Supplementary Figures***

**
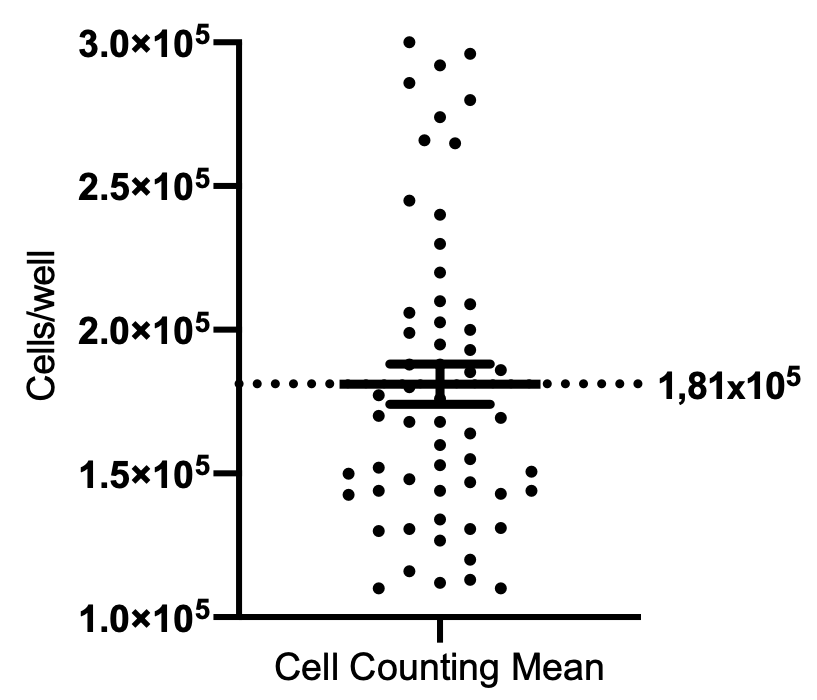
**

**Supplementary Figure 1. Mean number of the d-ODMs-cells/well counting before AIEC infection.** d-ODMs obtained from 7 different donors were plated in several replicates (n = 57) to determine the cell number at the time of infection.

**
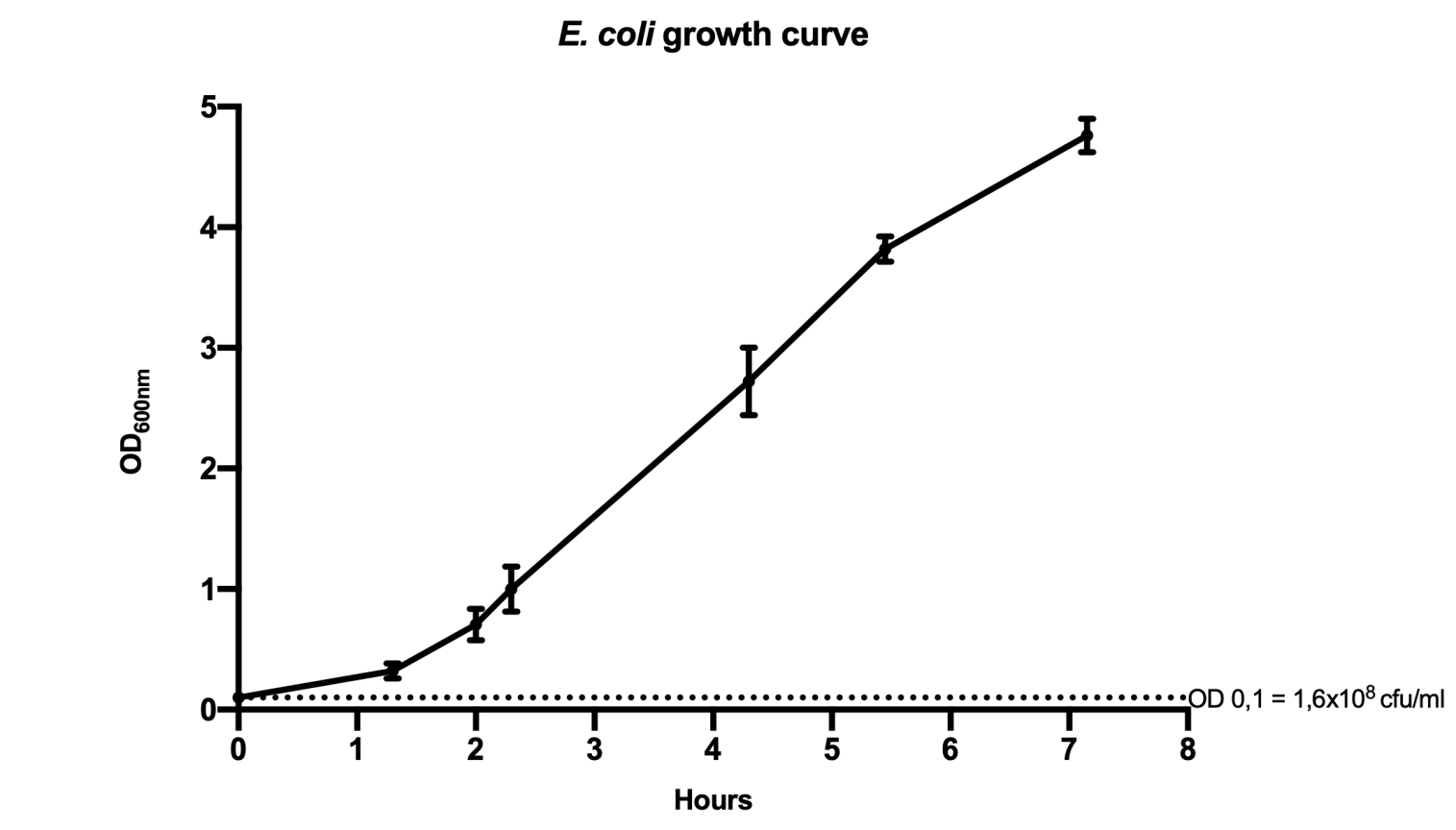
**

**Supplementary Figure 2. Mean value of the OD measurement and cfu/ml quantification of *E. coli* growth curve.** *E. coli* growth curve in LB was monitored for 7 hours by measuring the OD and by seeding the culture for cfu/ml quantification (n = 3 experimental replicates).

#

#

# Supplementary Figure 3. Gene expression analysis in ODMs and d-ODMs. Genes, which have been grouped according to functional classification, were analyzed by RT-qPCR to determine their expression levels in ODM vs. d-ODMs. A paired t-test was performed to examine statistically different expression patterns between the two groups (ODMs/d-ODMs; n = 5 for each culture type). A P value of <0.05 was considered statistically significant.

#
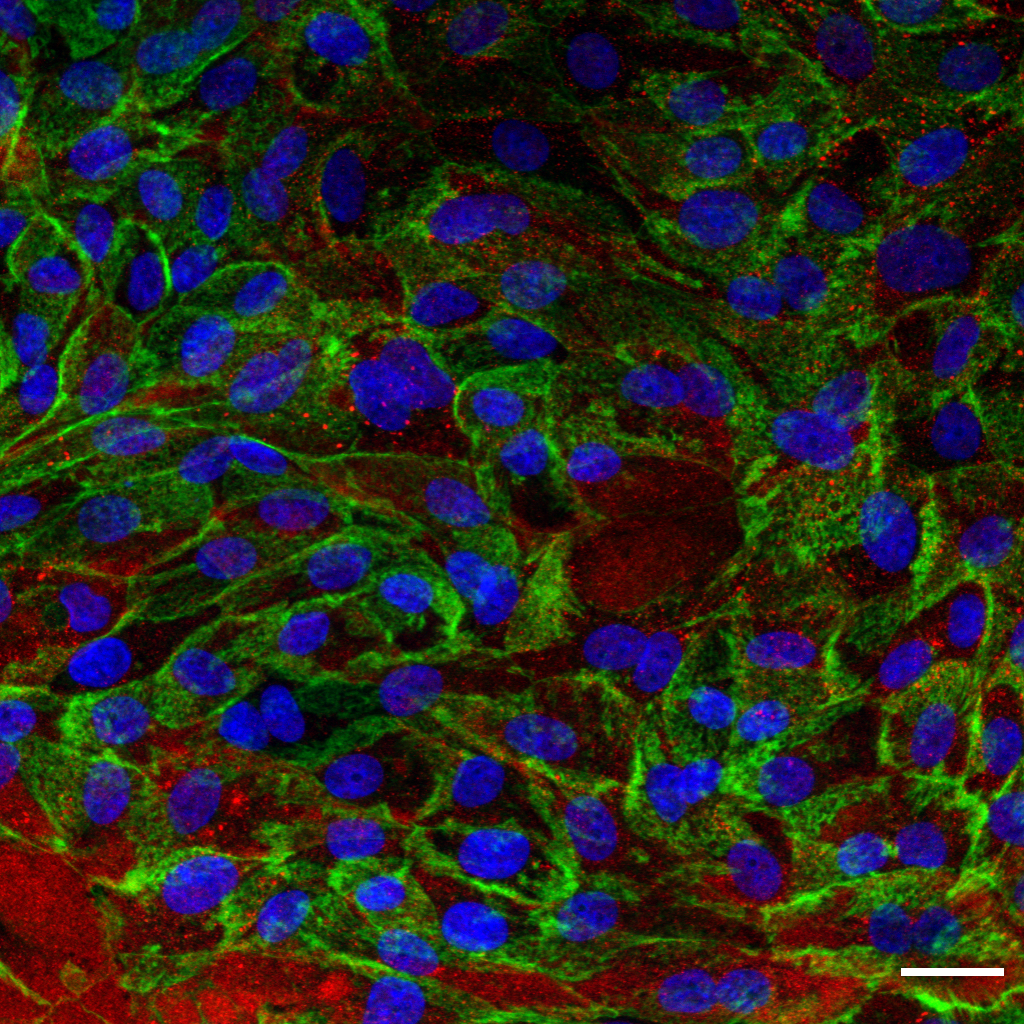

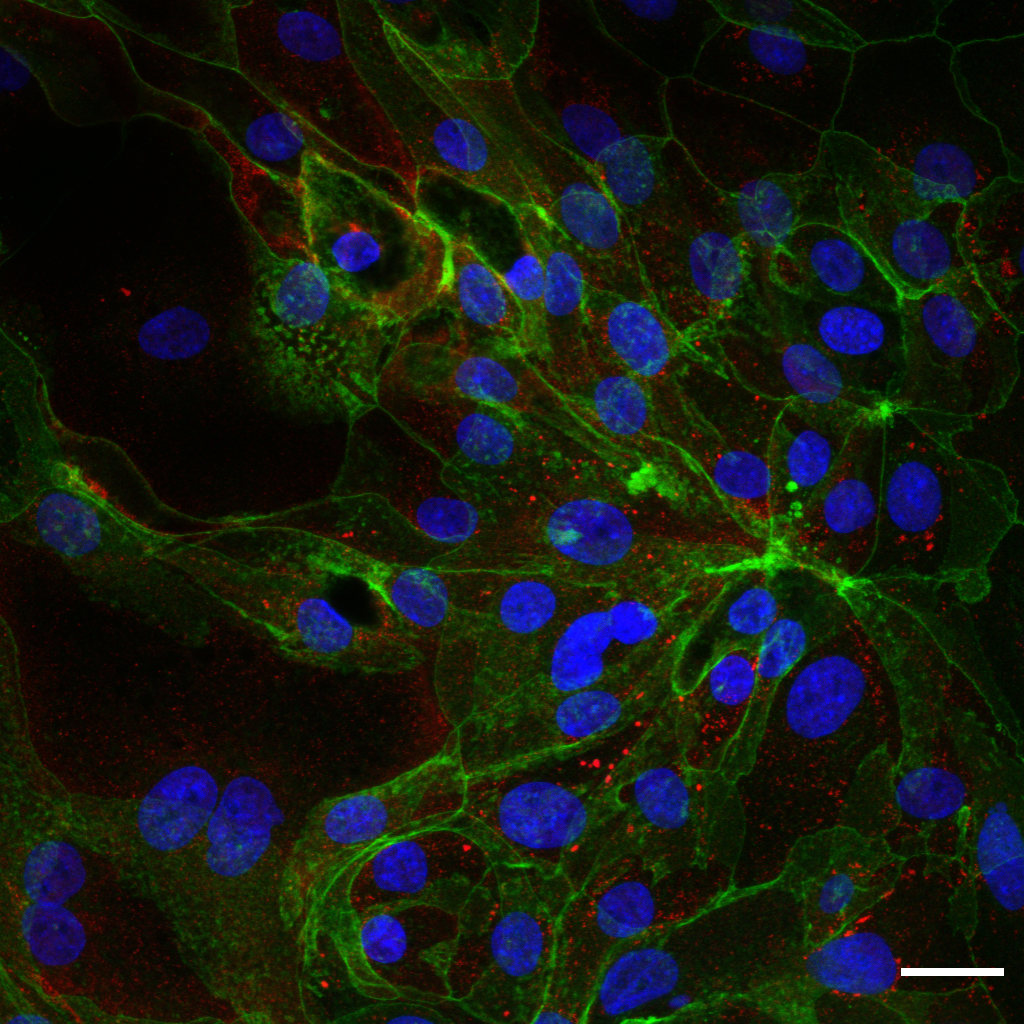


**A**

**ODMs d-ODMs**

**Villin/E-Cadherin/DAPI**

#

**B**

#

# Supplementary Figure 4. Protein expression analysis in ODMs and d-ODMs by immunofluorescence. (A) Villin (red stain), a cytoskeletal protein mainly expressed in the apical surface of the differentiated IECs, was analyzed to explore the differentiation status of ODMs vs d-ODMs. E-Cadherin, in green, was used as epithelial cell-wall marker. DAPI, in blue, counterstained the cell nuclei. Scale bars: 25 μm. Images are representative of n = 3 independent experiments performed using samples from two different donors. (B) Box-plot distribution of the fluorescence for the analyzed protein (Villin) in ODMs and d-ODMs, expressed as Mean Intensity. Fluorescence was quantified in 5 different fields per sample. A paired t-test was performed to examine statistically different expression patterns between the two groups (ODMs/d-ODMs). A P value of <0.05 was considered statistically significant.

**A**

#
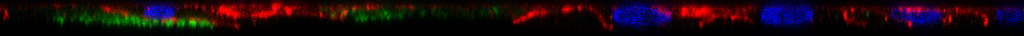


**ODM – MUC2/EpCAM/DAPI**

**d-ODM – MUC2/EpCAM/DAPI**

#
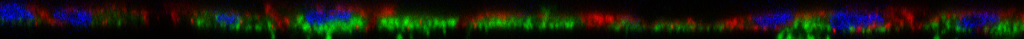


**B**

#
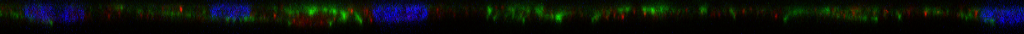


**ODM – VILLIN/E-Cadherin/DAPI**

**d-ODM – VILLIN/E-Cadherin/DAPI**

#
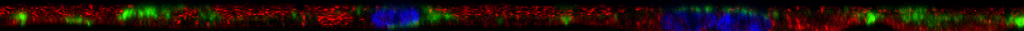


# Supplementary Figure 5. Orthogonal views of the protein expression in ODMs and d-ODMs analyzed by immunofluorescence. MUC2 (A) and Villin (B), both markers of differentiated IECs, were mainly expressed in the apical surface of d-ODMs compared to ODMs. EpCAM and E-Cadherin were used as epithelial cell-wall markers. DAPI counterstained the cell nuclei. Images are representative of n = 3 independent experiments performed using samples from two different donors.

#
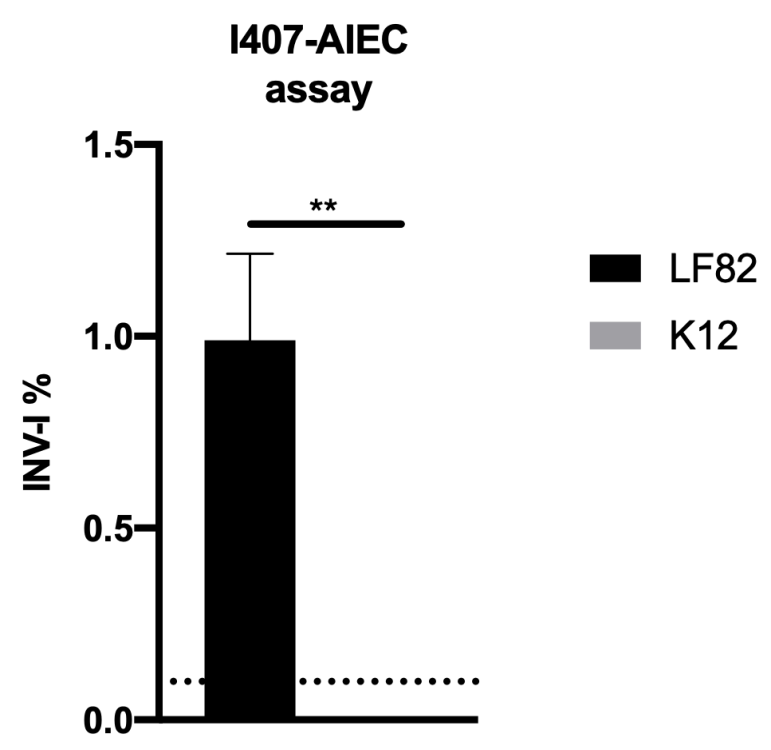


# Supplementary Figure 6. *E. coli* LF82 and K12 Invasion Indexes (INV-I %) in the I407 cell line. The dashed line represents the established threshold (0.1) that determines the invasive capacity of the *E. coli* strains tested in I407 cells (n = 5 experimental replicates). A paired t-test was performed to examine the statistical significance between LF82 and K12 INV-I %. A P value of <0.05 was considered statistically significant. ** indicates P = 0.0024.
