# Supplementary material for: A Novel Strategy to Study the Invasive Capability of Adherent-Invasive Escherichia coli by Using Human Primary Organoid-Derived Epithelial Monolayers
Source: Front Immunol. 2021 Mar 29;12:646906. doi: 10.3389/fimmu.2021.646906 (PMC8039293; doi:10.3389/fimmu.2021.646906)
Supplement: Supplementary file 2 [file DataSheet_2.docx]

***Supplementary Tables***

| **Supplementary Table 1. Clinical and demographic descriptions of the subjects from which ODMs and d-ODMs were obtained.** | | | |
| --- | --- | --- | --- |
| N | Age (years) | Gender (M/F) | Sample Type (Surgical/Biopsy) |
| Group 1 (ODMs/d-ODM culture: qPCR) | | | |
| 5 | 53-74 (55.6) | 3/2 | 4/1 |
| Group 2 (d-ODMs culture: AIEC infection) | | | |
| 7 | 53-69 (60.4) | 4/3 | 3/4 |
| Group 3 (ODMs/d-ODMs culture: Immunofluorescence) | | | |
| 6 | 53-72 (60.16) | 4/2 | 3/3 |

N: number of subjects included in the analysis.

Age: Range (mean).
qPCR: quantitative real-time RT-PCR

*For Supplementary Tables 2-9: Final concentrations (%, Molarity, dilutions (X)) are expressed relative to purchase concentration.

| **Supplementary Table 2. Washing medium composition** | |  |
| --- | --- | --- |
| **COMPONENT** | **MANUFACTURER** | **FINAL CONCENTRATION** |
| Advanced DMEM/F12 medium | Gibco, Grand Island, NY, USA  Ref. 12634-010 | 100% |
| HEPES | Gibco, Grand Island, NY, USA  Ref. 15630056 | 10mM |
| GlutaMAX | Gibco, Grand Island, NY, USA  Ref. 35050-038 | 1X |
| FBS | Gibco, Grand Island, NY, USA | 5% |

| **Supplementary Table 3. Dissociation medium composition** | |  |
| --- | --- | --- |
| **COMPONENT** | **MANUFACTURER** | **FINAL CONCENTRATION** |
| Advanced DMEM/F12 medium | Invitrogen, Carlsbad, CA, USA | 100% |
| HEPES | Gibco, Grand Island, NY, USA | 10 mM |
| GlutaMAX | Gibco, Grand Island, NY, USA | 1X |
| N-2 | Gibco, Grand Island, NY, USA | 1X |
| B-27 without retinoic acid | Gibco, Grand Island, NY, USA | 1X |
| Nicotinamide | Sigma-Aldrich, Saint Louis, MO, USA | 10 mM |
| N-Acetyl-L cysteine | Sigma-Aldrich, Saint Louis, MO, USA | 1 mM |
| Prostaglandin E2 (PGE2) | Sigma-Aldrich, Saint Louis, MO, USA | 2.5 μM |
| Y-27632 | Merck, Darmstadt, Germany | 10 μM |
| Dispase | Gibco, Grand Island, NY, USA Ref. 17105-041 | 400 μg/ml |

| **Supplementary Table 4. STEM+Y medium composition** | |  |
| --- | --- | --- |
| **COMPONENT** | **MANUFACTURER** | **FINAL CONCENTRATION** |
| Advanced DMEM/F12 medium | Gibco, Grand Island, NY, USA | 50% v/v |
| Wnt3a-conditioned medium | Produced using an L-Wnt3a cell line, ATCC CRL-2647 | 50% v/v |
| HEPES | Gibco, Grand Island, NY, USA | 10 mM |
| GlutaMAX | Gibco, Grand Island, NY, USA | 1X |
| N-2 | Gibco, Grand Island, NY, USA  Ref. 17502-048 | 1X |
| B-27 without retinoic acid | Gibco, Grand Island, NY, USA  Ref. 12587-010 | 1X |
| Nicotinamide | Sigma-Aldrich, Saint Louis, MO, USA  Ref. N0636-100G | 10 mM |
| N-Acetyl-L cysteine | Sigma-Aldrich, Saint Louis, MO, USA  Ref. A9165-25G | 1 mM |
| Normocin | InvivoGen, San Diego, CA, USA  Ref. ANT-NR-1 | 100 μg/ml |
| Gastrin I | Tocris Bioscience, Bristol, UK  Ref. 3006 | 1 μg/ml |
| Human Noggin | Peprotech, Rocky Hill, NJ, USA  Ref. 167120-10C-0100 | 100 ng/ml |
| Human Epidermal Growth Factor (EGF-1) | Gibco, Grand Island, NY, USA.  Ref. PHG0311 | 50 ng/ml |
| SB202190 | Sigma-Aldrich, Saint Louis, MO, USA  Ref. S7067 | 10 μM |
| LY2157299 | Axon MedChem, Groningen, the Netherlands  Ref. 1491 | 500 nM |
| R-Spondin-1 (RSPO1) | Sino Biologicals, Beijing, China Ref. 11083-H08H | 500 ng/ml |
| Prostaglandin E2 (PGE2) | Sigma-Aldrich, Saint Louis, MO, USA  Ref. P0409-1MG | 100 nM |
| Y-27632 | Merck, Darmstadt, Germany. Ref. 688000- 5MG | 10 μM |

| **Supplementary Table 5. DIFF medium composition** | |  |
| --- | --- | --- |
| **COMPONENT** | **MANUFACTURER** | **FINAL CONCENTRATION** |
| Advanced DMEM/F12 medium | Invitrogen, Carlsbad, CA, USA | 100 % |
| HEPES | Gibco, Grand Island, NY, USA | 10 mM |
| GlutaMAX | Gibco, Grand Island, NY, USA | 1X |
| N-2 | Gibco, Grand Island, NY, USA | 1X |
| B-27 without retinoic acid | Gibco, Grand Island, NY, USA | 1X |
| N-Acetyl-L cysteine | Sigma-Aldrich, Saint Louis, MO, USA | 1 mM |
| Normocin | InvivoGen, San Diego, CA, USA | 100 μg/ml |
| Gastrin I | Tocris Bioscience, Bristol, UK | 1 μg/ml |
| Human Noggin | Peprotech, Rocky Hill, NJ, USA | 100 ng/ml |
| Human Epidermal Growth Factor (EGF-1) | Invitrogen, Carlsbad, CA, USA | 50 ng/ml |
| LY2157299 | Axon MedChem, Groningen, the Netherlands | 500 nM |
| R-Spondin-1 (RSPO1) | Sino Biologicals, Beijing, China | 250 ng/ml |

| **Supplementary Table 6. Complete EMEM medium composition** | |  |
| --- | --- | --- |
| **COMPONENT** | **MANUFACTURER** | **FINAL CONCENTRATION** |
| Minimum Essential Medium – Eagle with Earle's BSS (EMEM) | Lonza, Basel, Switzerland  Ref. BE12-125F | 100% |
| FBS | Gibco, Grand Island, NY, USA | 10% |
| Antibiotic Antimycotic Solution | Sigma-Aldrich, Saint Louis, MO, USA  Ref. A5955 | 1% |
| MEM Vitamin Solution | Gibco, Grand Island, NY, USA  Ref. 11550386 | 1% |
| MEM Non-Essential Amino Acids Solution | Gibco, Grand Island, NY, USA  Ref. 11140035 | 1% |
| L-glutamine | Gibco, Grand Island, NY, USA  Ref. 11500626 | 1% |

| **Supplementary Table 7. EMEM-MM** | |  |
| --- | --- | --- |
| **COMPONENT** | **MANUFACTURER** | **FINAL CONCENTRATION** |
| EMEM | Lonza, Basel, Switzerland | 100% |
| FBS | Gibco, Grand Island, NY, USA | 10% |

| **Supplementary Table 8. DIFF-MM** | |  |
| --- | --- | --- |
| **COMPONENT** | **MANUFACTURER** | **FINAL CONCENTRATION** |
| Advanced DMEM/F12 medium | Invitrogen, Carlsbad, CA, USA | 100 % |
| HEPES | Gibco, Grand Island, NY, USA | 10 mM |
| GlutaMAX | Gibco, Grand Island, NY, USA | 1X |
| N-2 | Gibco, Grand Island, NY, USA | 1X |
| B-27 without retinoic acid | Gibco, Grand Island, NY, USA | 1X |

| **Supplementary Table 9. LB agar plates** (120x120mm square plates, (Corning, NY, USA)) | | |
| --- | --- | --- |
| **COMPONENT** | **MANUFACTURER** | **FINAL CONCENTRATION** |
| LB Broth | Lennox, Richardson, TX, USA | 16 g/L |
| Bacto Agar | BD Biosciences, San Jose, CA, USA. Ref. 10455513 | 20 g/L |
